# An interrupted time series analysis of the effect of the quality improvement team on the rate of adverse events in the Botswana safe male circumcision program from 2015 to 2019

**DOI:** 10.1371/journal.pone.0335587

**Published:** 2025-12-12

**Authors:** Ludo S. Monene, Billy M. Tsima, Keikantse Matlhagela, Mooketsi Molefi

**Affiliations:** 1 Department of Public Health and Family Medicine, Faculty of Medicine, University of Botswana, Gaborone, Botswana; 2 Department of Biomedical Sciences, Faculty of Medicine, University of Botswana, Gaborone, Botswana; Henry Jackson Foundation, UNITED STATES OF AMERICA

## Abstract

**Background:**

Voluntary medical male circumcision (VMMC) has been shown to reduce heterosexual human immune deficiency virus (HIV) transmission. In 2009, Botswana rolled out its VMMC program to expand existing HIV preventive strategies. However, in 2017, a study recorded an adverse event rate (AER) of 6.7% in Botswana. A quality improvement team was introduced to help reduce AER through standardized training and mentorship by ensuring that the World Health Organization VMMC and the Botswana safe male circumcision program standards are met. We hypothesized that **t**he introduction of the quality improvement team reduced the trend and magnitude of the moderate and severe AER for the day 7 routine follow-up visit.

**Methods:**

A quasi-experimental study was conducted using data from monthly district reporting tools. Interrupted time series analysis was used to compare the trend and magnitude of the moderate and severe AERs after circumcision for the day 7 routine follow-up visit in males aged 10 years and older. The comparison was done two years before (April 2015 to March 2017) and two years after (April 2017 to April 2019) the introduction of the quality improvement team. The most common adverse events (AEs) by age category, type, and severity between April 2015 and April 2019 were also reported. Frequencies and percentages were used to summarize the descriptive measures. Where indicated, all measures are reported with 95% confidence interval and statistical significance set at p ≤ 0.05.

**Results:**

There was an overall downward trajectory of the day 7 moderate and severe AER throughout the study period. Nonetheless, prior to the intervention, the moderate and severe day 7 AER decreased significantly monthly by 0.1% (p = 0.046, 95% CI = −0.195, 0.002). However, after the intervention, the AER insignificantly increased by 0.08% (p = 0.107, 95% CI = −0.018, 0.181). Majority (68.5%) of the AEs occurred in the 10–14 years age category. Most of these AEs were mild (73.8%). Infections were the most common AE (45.1%).

**Conclusion:**

The effect of the quality improvement intervention on the rate of AEs was minimal. Regular audits and further research on why the quality improvement team was not able to significantly reduce the AER would be beneficial.

## Introduction

Voluntary medical male circumcision (VMMC) has been proven to be a highly effective intervention for preventing heterosexual human immunodeficiency virus (HIV) transmission, as it offers lifelong partial protection against HIV infection [1–5]. In 2007, the World Health Organization (WHO) recommended that countries with high a HIV burden and low circumcision prevalence should upscale circumcision services [5]. This decision followed studies carried out in three countries in Africa, which showed that male circumcision could reduce the risk of heterosexual HIV transmission by approximately 60% [2–5]. The formation of the Safe Male Circumcision (SMC) program by the Ministry of Health (MoH) in Botswana in 2009 followed the WHO recommendations [6]. Male circumcision in the country is mainly performed for HIV prevention, but it can be performed when there are other medical indications regardless of the client’s HIV status. The procedure is done in all districts at different facilities like health posts, mobile and static clinics and hospitals. Some of the districts are supported by implementing partners such as the African Comprehensive HIV/AIDS Partnerships (ACHAP) and the Johns Hopkins Program for International Education in Gynecology and Obstetrics (Jhpiego). However, documentation and reporting to MoH is overseen by the district SMC focal person. After the procedure, there are three scheduled routine follow-ups at day two, day seven and day forty-two. Nonetheless, if concerns or complication arise, clients are reviewed on any other day. Botswana transitioned from actively performing VMMC for males aged 10 years and older to males aged 15 years and above in February 2021 following the 2020 WHO and the U.S. President’s Emergency Plan for AIDS Relief (PEPFAR) guidance [7]. The VMMC uptake in Botswana began slowly and remained modest with less than 50% of the national target achieved in 2018 [6]. This could be attributable to the unwillingness of men to undergo the procedure secondary to concerns about potential risks [8].

An adverse event (AE) is any injury, harm or undesired outcome that occurred during or following male circumcision that would not have occurred if the client had not undergone the procedure [9]. Adverse events may occur because of patient, provider and community factors, and may not be related to any error, clinical experience or expertise [3,4]. AEs have been reported in VMMC programs, including in Botswana [2–4,6]. In 2007, the rates of complications in Africa were suggested to be poorly documented in clinical settings but could vary from 2% to as high as 17.5% in research settings [3]. However, a meta-analysis including studies from across 10 countries in Africa showed that the proportion of AEs ranged from 0.70% to 37.36% [10]. Adverse event rate (AER) is the proportion of circumcised males with AEs out of all circumcised males who came for at least one post-circumcision follow-up visit [9]. Studies carried out in the eastern and southern parts of Africa have also demonstrated varying AERs [11–16]. The discordancy in AERs could be due to varying classifications of AEs across settings and the differences in post-circumcision follow-up rates reported in the studies. Higher retention rates (number of males who come for at least one post-circumcision follow-up visit) indicated that most circumcised males were reviewed after the procedure. This suggests that most of the AEs that occurred were likely to be identified and documented if clients honoured their post-circumcision review appointments. Nonetheless, AEs that presented earlier or later than the follow-up date were likely to be undetected. Notably, in southern African countries, the majority of reported AERs fell within the WHO acceptable level of 2% [14,15]. However, these studies suffered poor retention rates hence a poor reflection of the AERs. A prospective cohort study by Wirth *et al* estimated a rate of 6.7% for moderate and severe AEs and a retention rate of 97.2% for at least one follow up visit within 7 days after circumcision in adult men at two government clinics in Gaborone, Botswana. The most common AE was hematoma [16]. The observed high retention rate in this study possibly contributed to the observed high AER. Similarly, with 94.2% of clients contributing to follow-up and moderate or severe AE data, Reed *et al* documented a higher AER of 6.8% in clients who were lost to follow-up and reviewed approximately two weeks post-surgery compared to a rate of 3.3% for those who were adherent to follow-up in 13–14 days after surgery [13].

When WHO recommended male circumcision as part of a comprehensive HIV prevention package in 2007, countries were also advised to ensure that training and certification of service providers are rapidly implemented to increase the safety and quality of services [5]. SMC programs should have a plan to maintain high quality pre and post procedure services, ensuring regular monitoring of AEs [17–20]. Some studies have shown that the quality improvement process does not significantly change the moderate and severe AERs [21]. However, others have shown that the overall quality of services including the rate of moderate plus severe AEs reduced significantly with the quality improvement approach [22,23]. These studies had limitations such as non-immunity to secular changes and data quality issues, which could lead to over or underestimation of the rate of AEs.

In Botswana, the national continuous quality improvement (CQI) team was introduced by the MoH in 2017 and was supported by PEPFAR through the Center for Disease Control and Prevention (CDC). Members of the team included the national program coordinator, MoH program officer, MoH monitoring and evaluation officer, district level health education officers and clinicians (medical officers and nurses). The team initially comprised of four clinicians from MoH whose core duties also included providing health care that is not related to male circumcision in their respective districts. It is unclear how the numbers changed over the years and MoH had no capacity to enrol CQI teams on a permanent basis. The purpose of the CQI team was to offer standardized training, support and mentorship to healthcare workers on demand creation, service delivery and monitoring and evaluation of male circumcision across all districts in the country. This included training on the male circumcision procedure and identification, classification, management and reporting of AEs. It also included demand creation activities and guidance on documentation of facility-based registers and reporting tools. Training material was developed by MoH, Jhpiego and ACHAP. Training and mentorship comprised of both theory (two days didactic) and practical (three days theatre work) which could either be done centrally or at district level depending on availability of the CQI team members, resources and clients. Ongoing mentorship, which varied according to the level of competency of the trainees and district needs, was also offered prior to certification. Only trained and certified personnel were allowed to carry out the procedure, but sensitized personnel at any facility could create demand and follow-up clients.

Data on the rates of AEs and their trends with quality improvement interventions within VMMC programs in Africa exists, specifically in countries with a high HIV burden and low circumcision prevalence. However, data that describes the trend and magnitude of AEs in relation to continuous quality improvement using multiple measurements to evaluate the safety of VMMC programs are limited. The purpose of this study was to assess whether the introduction of the quality improvement team into the SMC program changed the rate of moderate and severe AEs for the day 7 routine follow-up visit between 2015 and 2019 in males aged 10 years and older in Botswana. The study also identified the most common AEs by age category, severity and type.

## Methods

### Study design

Monthly reports were reviewed to analyse the trends and magnitude of moderate and severe AERs for the day 7 routine follow-up visit in males aged 10 years and older undergoing SMC before and after the introduction of the CQI team. This best captures the possible reported AEs, and it is comparable with other studies. All moderate and severe AEs recorded for the day 7 follow-up visit were used to calculate the monthly AERs which were then used as data points between 1^st^ April 2015 and 30^th^ April 2019. A total of 48 consecutive data points of aggregated monthly AERs were used, with 24 data points being before the CQI intervention (1^st^ April 2015–31^st^ March 2017) and the other 24 data points being after the CQI intervention (1^st^ April 2017–30^th^ April 2019). The interruption point was April 2017, when the quality improvement team was introduced.

### Main variables measured

Age category, method of circumcision (surgical or PrePex), HIV status (negative, positive or unknown), SMC counselling (done/not done), districts (rural or urban), follow up visits, types and severity of AEs were measured and available for the analysis. The Adverse Event Action Guide by Population Services International and College of Surgeons of East, Central and Southern Africa was used by healthcare providers to identify and classify AE severity [9]. The AER, which is the proportion of circumcised males with AEs out of all circumcised males who came for the day 7 post-circumcision follow-up visit, was calculated. The retention rate, which is the proportion of circumcised males who came for the day 7 follow-up visits out of all circumcised males, was also calculated [9].

### Data sources, collection and extraction

Quantitative data were collected from the monthly reporting tools that are sent to MoH by each of the 27 District Health Management Teams (DHMTs) in Botswana. Districts report these data manually, but those with access to the internet have the option to report the data electronically through the District Health Information Software 2 (DHIS 2) in the same reporting tool. From these reports, the total number of males circumcised, the total number of those with AEs on day 7, and those who came for the day 7 routine follow-up were extracted and inserted into tables designed by the researchers. These data were then used to calculate the rate of moderate and severe AEs and the retention rates. The types and severity of AEs as well as age categories were extracted for the analysis. Additional information included HIV status (positive, negative or unknown), whether counselling was done before the procedure or not, the method of circumcision used (surgical or PrePex) and whether circumcision was done in an urban or rural facility.

Records of all circumcised males aged 10 years and older were included regardless of the type of procedure (surgery or PrePex device) used for the circumcision. Records with missing data for specific variables (facility and district name, reporting month or year, age groups, number of clients counselled, HIV status, number of SMC conducted, number of clients who came for review, AEs and severity) were excluded. All recorded data from the monthly reports were accessed between 4^th^ February 2021 and 4^th^ February 2022.

### Data processing and analysis

Interrupted time series analysis was used. Data extracted from the district reporting tools were initially entered into dummy tables in Microsoft excel and AERs were calculated for each month. Analysis was done using Stata version 13 software (Statacorp, College Station, TX, USA). In Stata, data were declared to be time series and were sorted by time, where the time variable was set as month. The day 7 AEs were then regressed using the Newey model which estimates coefficients by ordinary least squares regression but produces Newey-West standard errors to handle autocorrelation in addition to possible heteroskedasticity. Without a comparison group, the standard interrupted time series analysis regression model assumes the following form:

Y_t_ = β_0_ + β_1_T_t_ + β_2_X_t_ + β_3_X_t_T_t_ + ∈_t_ where:

Y_t_ = aggregated outcome variable measured at each equally space time point t

T_t_ = time since the start of the study

X_t_ = dummy (indicator) variable representing the intervention (pre-intervention 0, otherwise 1)

X_T_T_t_ = an interaction term

β_0_ = intercept or starting level of the outcome variable (AER)

β_1_ = slope or trajectory of the outcome variable (AER) until the introduction of the intervention (CQI team)

β_2_ = change in the level of the outcome that occurs in the period immediately following the introduction of the intervention (compared with the counterfactual)

β_3_ = difference between the pre-intervention and post-intervention slopes of the outcome

∈_t _= first-order autoregressive process when the random error terms occurs

Time series scatter plots were created. The *actest* was used to test for autocorrelation. P-value was conventionally set at ≤0.05 to indicate the presence of statistical significance and precision at 95% confidence interval. Significant p- values in β_3_ were looked for to indicate the effect of the CQI team on the moderate and severe AER over time. The design assumes that any time varying confounder changes relatively slowly. With multiple measurement for both the pre- and post-intervention period, it was easier to control for confounding factors and overcome spurious detection of effects that result from underlying secular trends of the data such as change and shifts in guidelines, rolling out of guidelines and cultural norms such as the belief that doing the procedure during winter is better than during summer, which results in higher client volumes in winter. The analysis allows for autocorrelation between outcomes. However, there is need to use the design with caution when there are multiple policy changes around the point of intervention, or when the intervention occurred prior to the true or actual point of intervention [24]. Furthermore, the proportion of AEs that occurred within the specified period was profiled by age groups (10–14 years, 15–19 years, 20–24 years, 25–29 years, 30–39 years, 40–49 years and 50 years or older), type (anaesthetic reaction, bleeding, haematoma, infection, swelling, gaping and others) and severity (mild, moderate, severe) to identify the most common of these.

### Ethical clearance

Ethical approval was granted by the University of Botswana Institutional Review Board (UBR/RES/IRB/BIO/GRAD/126) and additionally by the Health Research and Development Committee at the Ministry of Health in Botswana (HPDME I3/I8/I). Further approval to access data was sought from the safe male circumcision program (HSM 6/9/2 I). Participant consent was not obtained because no personal information is captured in the data collection tool. Thus, all data extracted from the reports and analysed for this study were fully anonymised before access by researchers. The authors did not have access to information that could identify individual participants during or after data collection. Data obtained were used for research purposes only, with access restricted to the researchers.

## Results

**Fig 1:**
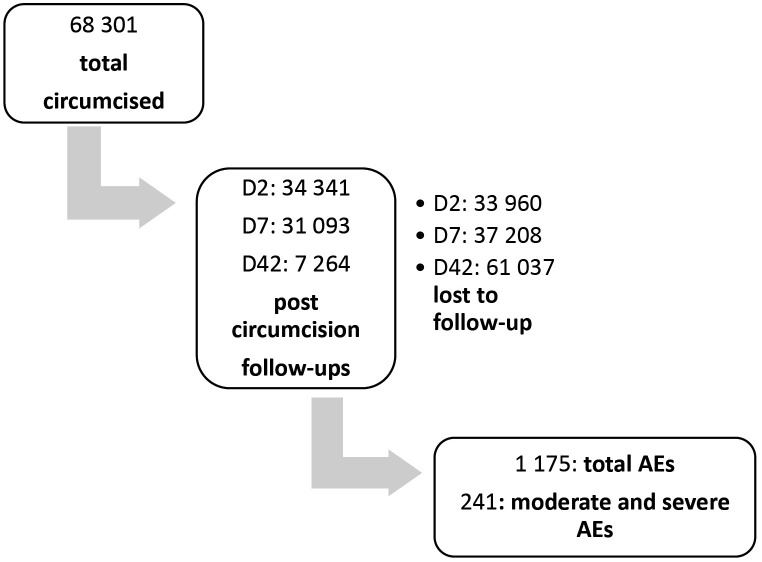
Flow diagram showing the total number circumcised, followed up and the total number of AEs recorded during the study period. a) D2: day 2, b) D7: day 7, c) D42: day 42.

A total of 15 230 district monthly reports were reviewed of which 13 (11 from the pre-intervention and 2 from the post-intervention period) were excluded because they had incomplete data for specific variables. A total of 322 reports were missing: 248 were from the pre-intervention period and 74 were from the post-intervention period. Between the 1^st^ of April 2015 and the 30^th^ of April 2019, a total of 68 301 males aged 10 years or more were circumcised in the Botswana SMC program, Fig 1. Of these males, only 34 341 (50.3%), 31 093 (45.5%) and 7 264 (10.6%) came back for the day 2, day 7 and day 42 scheduled follow-up visits, respectively. The remainder were lost to follow-up, i.e.,33 960 (49.7%), 37 208 (54.5%), 61 037 (89.4%) for the scheduled day 2, day 7 and day 42 follow-up visits respectively. A total of 1 175 AEs were identified during this period and 241 of these were in the moderate and severe category. These results are illustrated in Fig 1.

The demographic and clinical characteristics of participants are presented in Table 1. Most of the males that were circumcised were in the 10–14 years age group with 44 835 (65.4%) circumcisions and lowest was in the 50 + years age group with 748 (1.1%) circumcisions. A total of 46 474 (68%) of these circumcisions were conducted in rural districts. The method of circumcision was not specified for most patients, 67 712 (99.1%). However, for those whose method of circumcision was specified, the majority were surgically circumcised. A significant proportion of males were HIV negative, 51 722 (75.7%), whilst 1 784 (2.6%) were HIV positive and 14 795 (21.7%) had an unknown HIV status. Counselling prior to the procedure was performed for most of the clients, 67 718 (99.1%).

**Table 1:** Characteristics of circumcised clients from April 2015 to April 2019.

Characteristics	Circumcised clients N = 68301 n (%)
Age category in years	
10-14	44 835 (65.6)
15-19	6 239 (9.1)
20-24	5 758 (8.4)
25-29	4 817 (7.1)
30-39	4 380 (6.4)
40-49	1 524 (2.2)
50+	748 (1.1)
Method of circumcision	
Surgical	511 (0.8)
PrePex	78 (0.1)
Unspecified	67 712 (99.1)
HIV status	
Negative	51 722 (75.7)
Positive	1 784 (2.6)
Unknown	14 795 (21.7)
Counselled	
Yes	67 718 (99.1)
No	0
Not specified	583 (0.9)
Districts	
Urban	21 827 (32.0)
Rural	46 474 (68.0)

The types of AEs reported for participants stratified by age category are presented in Table 2. Many of the AEs were recorded within the 10–14 years age group, and the least AEs were in the 50 + years age group with 682 (58.0%) and 10 (0.1%), respectively. Two of the clients’ ages were not specified. Infection was the commonest AE and surgical wound gaping was the least common with 530 (45.1%) and 48 (4%), respectively. Other AEs included 5 (0.4%) delayed healing, 7 (0.6%) mild pain, 2 (0.2%) excessive skin removal, 7 (0.6%) insufficient skin removal, 1 (0.1%) self-induced burning, 3 (0.2%) PrePex removal and 41 (3.5%) AEs not otherwise specified.

**Table 2:** Types of AEs stratified by age category (n = 1175).

Age Category (years)/ AE	Anaesthetic reaction n (%)	Bleeding n (%)	Hematoma n (%)	Infection n (%)	Swelling n (%)	Gaping n (%)	Others n (%)	Total n (%)
10-14	36 (5.3)	115 (16.9)	110 (16.1)	363 (53.2)	17 (2.5)	10 (1.5)	31 (4.5)	682 (58.0)
15-19	3 (3.2)	16 (17.2)	13 (14.0)	40 (43.0)	11 (11.8)	2 (2.2)	8 (8.6)	93 (7.9)
19-24	22 (14.5)	30 (19.7)	19 (12.5)	46 (30.3)	20 (13.2)	9 (5.9)	6 (3.9)	152 (12.9)
25-29	0	14 (15.2)	12 (13.0)	37 (40.2)	9 (9.8)	12 (13.0)	8 (8.7)	92 (7.8)
30-39	5 (4.5)	28 (25.5)	9 (8.2)	30 (27.3)	16 (14.5)	13 (11.8)	9 (8.2)	110 (9.4)
40-49	2 (5.9)	5 (14.7)	9 (26.5)	12 (35.3)	3 (8.8)	0	3 (8.8)	34 (2.9)
50+	0	5 (50.0)	1 (10.0)	2 (20.0)	1 (10.0)	1 (10.0)	0	10 (0.9)
Age unknown	0	0	0	0	0	1 (50)	1 (50)	2 (0.2)
Total	68 (5.8)	213 (18.1)	173 (14.7)	530 (45.1)	77 (6.6)	48 (4.1)	66 (5.6)	1175

The severity of AEs reported, and the sum of moderate and severe AEs stratified by age category are presented in Table 3. Overall, most of the AEs were mild and the least common were the severe ones with 868 (73.8%) and 48 (4.1%) respectively across all age categories. Combined, the moderate and severe AEs were more common in the 10–14 years age group with 137 (56.9%) AEs recorded. Severity was not classified in 66 (5.6%) of the documented AEs.

**Table 3:** Severity of AEs stratified by age category (n = 1175).

Age Category (years)/ Severity	Mild n (%)	Moderate n (%)	Severe n (%)	Moderate & Severe AEs n (%)	Unclassified n (%)
10-14	514 (75.4)	108 (15.8)	29 (4.3)	137 (56.9)	31 (4.5)
15-19	67 (72.0)	14 (15.1)	4 (4.3)	18 (7.5)	8 (8.6)
19-24	119 (78.3)	21 (13.8)	5 (3.3)	26 (10.8)	7 (4.6)
25-29	58 (63.0)	19 (20.7)	4 (4.3)	23 (9.5)	11 (12.0)
30-39	75 (68.2)	25 (22.7)	4 (3.6)	29 (12.0)	6 (5.5)
40-49	24 (70.6)	6 (17.6)	2 (5.9)	8 (3.3)	2 (5.9)
50+	10 (100)	0	0	0	0
Age Unknown	1 (50)	0	0	0	1 (50)
TOTAL	868 (73.9)	193 (16.4)	48 (4.1)	241 (100)	66 (5.6)

Results of regression analyses of the day 7 moderate and severe AERs (D7AER) with Newey-West standard errors are presented in Table 4. The D7AER at the beginning of the study period was 2.95%. Prior to the intervention, the trend shows that the D7AER significantly fell every month by a magnitude 0.1% (p = 0.046, 95% CI = −0.195, 0.002), while they insignificantly increased by 0.08% (p = 0.107, 95% CI = −0.018, 0.181) during the post intervention period. Notably, in the first month of the intervention, there appeared to be an insignificant increase in the D7AER of 0.26% (p = 0.585, 95% CI = −0.685, 1.201). The linear combination parameters (lincom) estimate produced by specifying the post-intervention trend shows that after introducing the CQI team, the D7AER insignificantly decreased monthly at a rate of 0.02% (p = 0.136, 95% CI = −0.04, 0.01).

**Table 4:** D7AER regressions with Newey-West standard errors.

PERIOD	D7AER		
	Coefficient (%)	p- value	CI (%)
**Starting level of D7AER** **(April 2015)**	0.0294941 (2.95)	0.002	0.0117318 (1.173), 0.0472563 (4.726)
**Pre intervention Period** **(April 2015-March 2017)**	− 0.000987 (0.1)	0.046	−0.0019555 (0.195) −0.000018 (0.002)
**Intervention Month** **(April 2017)**	0.0025775 (0.26)	0.585	−0.0068585 (0.685) 0.0120135 (1.201)
**Post intervention period** **(April 2017-April 2019)**	0.0008133 (0.08)	0.107	−0.0001823 (0.018) 0.0018089 (0.181)
**Post intervention linear trend** **(April 2017 to April 2019)**	− 0.0002 (0.02)	0.1361	−0.0004 (0.04), 0.0001 (0.01)

**Fig 2:**
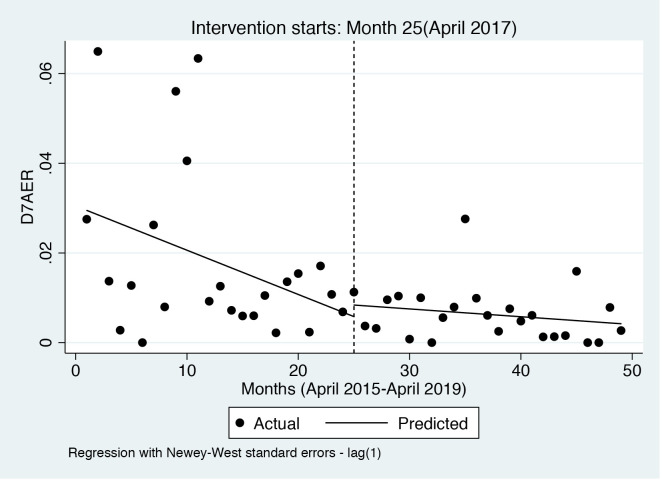
D7 AER trend.

The visual display of the results is presented in Fig 2.

## Discussion

In this study, there was an overall downward trajectory of the day 7 moderate and severe AERs throughout the study period. Nonetheless, prior to the intervention, they decreased significantly monthly by 0.1% and insignificantly increased by 0.08% during the post intervention period. These results showed that there was no overall significant reduction in the AERs after the CQI intervention. Similarly, a study conducted in 2015 in Uganda using secondary data, and with comparable retention rates, showed no significant change in moderate and severe AERs post the quality improvement intervention [21]. However, in Tanzania, a study indicated that with the CQI process, the rate of the AEs reduced, and the retention rates improved [25]. Comparably, a quasi-experimental study in Malawi highlighted a 48% reduction in AERs [23]. The reason for these results could be because the CQI intervention was more robust with a clear mandate and plans that went down from national to facility level. In our setting, districts largely had trained personnel and CQI support was mostly offered to DHMT’s on demand basis and availability of CQI team members who were healthcare workers obliged to offer other clinical duties outside of the circumcision program. The CQI was funded through MoH and the exact number of the CQI team members is unclear, but it changed over time and availability of the members also varied.

From our study, majority of circumcisions were recorded in the 10–14 year age group and some settings have also observed the same [26]. Most (58%) of the AEs occurred in the 10–14 years age category. Comparably, various studies conducted in the region have shown that most of the AEs occur in the same group. In Mozambique and Zimbabwe, the AE proportions were 80%, and 38% respectively in the 10–14 year age category [27,28]. A different study done in Zimbabwe in 2019 showed that 81% of patients with AEs were in the same age category while a cross-sectional study carried out in 14 countries showed an overall proportion of 45% [26,29]. These discrepancies may be a reflection of the existence of varying retention rates, surveillance systems and varying guidelines and methods of circumcision across settings, which carry different types and risks for AEs [15,30]. Another reason for the discrepancy could be, that clients in the 10–14 years age category are mostly dependent on their caregivers, therefore, identification of the AEs is not always accurate.

The most common AE in this study was infection, accounting for 45% of all AEs. Similarly, in Zimbabwe and Namibia, infections were the most common type of AE and they occurred commonly in the 10–14 year category [15,31]. Adolescents have been shown to be more at risk for infection because of poor post-operative care which is mostly a consequence of non-conformation to available program guidelines [15,32–35].

Most of the AEs in our study were mild (73.8%), with the severe ones being the least common (4.1%). Moderate AEs were 16.4% and the severity of 5.6% of all AEs was not specified. Whilst there is a paucity of data with respect to mild AEs, studies have shown that severe AEs are generally rare compared to moderate AEs [28,29,31]. Our study showed that moderate and severe AEs occurred mostly in the 10–14 years age category. This could be because majority of the circumcisions performed during the study period were in this age category. Similar studies have shown that moderate and severe AEs were more likely to be experienced by clients who are aged 10–14 years [30,36,37]. Nonetheless, a cross-sectional study across 14 countries in the region showed that AERs amongst the 10–14 year old males were higher compared to older age categories, but there was no significant association between the age and severity [26]. This discrepancy could be due to the fact that the studies that showed an association between age and AE severity were case series that focused on AEs that were not routinely reported or could potentially be missed like glans injuries and fistulas [28].

In this study, overall retention rates were low, with day 2, day 7 and day 42 having 50.3%, 45.5% and 10.6% of all circumcised clients coming back for routine follow-ups respectively. We could not verify which client came for which follow-up visit. Studies have documented varying retention rates ranging from 18% to 99% for day 2 and day 7 routine follow-ups [25,38]. Higher retention rates are desirable because they indicate that more circumcised males come for scheduled follow-up visits after the procedure, hence potential identification of AEs. Interestingly, the retention rates across different settings have remained inconsistent over the years [26]. This can be accounted for by factors which can be unique to the different settings like varying follow-up systems and poor follow-up secondary to geographical location. Some of the clients may be in hard-to-reach places especially in rural areas. In our study, 68% of the circumcised clients were in rural settings. Some of the clients do not come for follow up because of VMMC related stigma [32,34,35]. Pressure to reach targets which results in short or no counselling and poor documentation have been shown to result in low retention rates [26,39]. In our study, the reporting tool had missing data for some of the variables and some of reports could not be located. This suggests substandard documentation and data management which could have contributed to the low retention rates. Although the method of circumcision was not specified for most of the clients in our study, device based circumcisions have been shown to represent a small percentage of circumcision methods [26,40–42]. In Botswana PrePex represented a small percentage of circumcision methods and it was not rolled out in all districts. PrePex circumcisions dropped from 28% between 2015 & 2016 to 0% in 2017 [26]. In our study, only one out of the 27 districts documented the use of PrePex.

The strength of this study is that it is novel in the Botswana setting. Gaps within the SMC program such as low retention rates and poor documentation were also identified. The analysis used data from a large national registry and the level of missing data in the study was low, thus improving the validity of the analysis. Multiple data points were available for both pre- and post-intervention to assess trends, therefore, it was easier to address and control for confounding factors.

The limitations of this study were that secondary data were used so its validity could not be fully ensured. Training, support and mentorship by the CQI team was dependent on the availability of resources and client, therefore, it is unclear if all the districts were supported equally. Additionally, no clinic level data was reviewed to assess undocumented or misclassified AEs. Furthermore, some of the monthly reports could not be located. The reporting tools did not have all the information relating to AEs like method of circumcision, when the AEs occurred in relation to the procedure, explanations of some of the recorded AEs which did not have standard definitions or how clients who came for follow-up outside the routine visits were accounted for. Thus, AEs could possibly be underreported. The data available for our analysis did not include clients who did not come back for any post circumcision follow-up visit, thus potentially introducing residual bias as these may have been characteristically different from those included in the analysis. However, a recognized advantage of interrupted time series analysis is that groups or individuals serve as their own controls thereby reducing bias due to in-between group differences [24,43]. Low retention rates in the study could have also resulted in the underestimation of the reported AERs and more importantly, masking of the changes in AERs over time.

## Conclusion

There was an overall downward trajectory in the D7AER throughout the study period. However, there is need to study the fidelity of the quality improvement team through audits and further research to establish why there is no significant changes in the trend and magnitude of the D7AER between the pre and post CQI intervention periods. Gaps identified like poor documentation and low retention rates should be targeted for improvement. There is need to consider alternative follow-up methods such as engagement of community healthcare workers and use of telemedicine. The current reporting tool should be updated to capture some of the important data that can assist in fully assessing the SMC program in Botswana.

## Supporting information

S1 Dataset Day seven moderate and severe adverse event and retention rates. (XLSX)
